# Geschlechterunterschiede bei der posttraumatischen Belastungsstörung: aktuelle Evidenz zu Entstehung, Verlauf und Behandlung

**DOI:** 10.1007/s00115-025-01907-6

**Published:** 2025-10-06

**Authors:** Stephanie Haering, Caroline Meyer, Christine Knaevelsrud, Sinha Engel

**Affiliations:** 1 Klinisch-Psychologische Intervention, Freie Universität Berlin, Habelschwerdter Allee 45, 14195 Berlin, Deutschland; 2 Klinische Psychologie und Psychotherapie, Philipps-Universität Marburg, Marburg, Deutschland; 3 Deutsches Zentrum für Psychische Gesundheit, Berlin-Potsdam, Deutschland; 4 Klinische Psychologie und Psychotherapie, Freie Universität Berlin, Berlin, Deutschland; 5 Klinische Psychologie und Behavioral Health Technology, Friedrich-Alexander-Universität Erlangen-Nürnberg, Erlangen, Deutschland

**Keywords:** Trauma, Risikofaktoren, Geschlecht, Gender, Diversität, Trauma, Risk factors, Sex, Gender, Diversity

## Abstract

**Hintergrund:**

Geschlechterunterschiede bei psychischen Störungen sind weit verbreitet. Die posttraumatische Belastungsstörung (PTBS) zählt hierbei zu den Störungen mit den größten Prävalenzunterschieden zwischen Männern und Frauen.

**Ziel der Arbeit und Methode:**

Dieser narrative Übersichtsartikel beleuchtet die aktuelle wissenschaftlichen Evidenz zu Geschlechterunterschieden in Entstehung, Diagnostik und Behandlung der PTBS.

**Ergebnisse:**

Obwohl Männer häufiger traumatische Ereignisse erleben, haben Frauen ein zwei- bis dreifach höheres Risiko, an einer PTBS zu erkranken, und zeigen schwerwiegendere PTBS-Symptome als Männer. Die erhöhte Vulnerabilität von Frauen ist bisher nicht vollständig verstanden und auf eine Kombination aus biologischen und psychosozialen Faktoren zurückzuführen. Trotz ihres erhöhten Risikos sind Frauen sowie frauenspezifische Risikofaktoren in der relevanten Forschung unterrepräsentiert – es besteht ein Gender-Data-Gap. Männer erleben häufiger akzidentielle und bewaffnete Traumata, Frauen erleben häufiger sexuelle Gewalt. Während Frauen häufiger an komorbiden Angst- und affektiven Störungen leiden, tritt eine PTBS bei Männern vermehrt in Kombination mit Substanzmissbrauch auf. Männer mit einer PTBS nehmen seltener psychotherapeutische Hilfe in Anspruch als Frauen und profitieren weniger gut von evidenzbasierten traumafokussierten Interventionen.

**Diskussion:**

Die differenzierte Betrachtung biologischer und psychosozialer Faktoren ist entscheidend, um Geschlechterunterschiede in der PTBS zu verstehen. Geschlechtersensible Ansätze in Diagnostik und Behandlung sowie die Berücksichtigung von Geschlecht über binäre Klassifikationen hinaus können helfen, Wissenslücken zu schließen und eine gezieltere Versorgung zu ermöglichen.

**Zusatzmaterial online:**

Die Online-Version dieses Beitrags (10.1007/s00115-025-01907-6) enthält Informationen zu Geschlechteraspekten in der biologischen Stressreaktion.

## Abstract (en)

**Background:**

Sex and gender differences in mental disorders are widespread. Posttraumatic stress disorder (PTSD) is one of the mental disorders with the largest prevalence differences between women and men.

**Aim of the paper and methods:**

This narrative review article highlights the current scientific evidence on sex and gender differences in the development, diagnostics and treatment of PTSD.

**Results:**

Although men more frequently experience traumatic events women have a twofold to threefold higher risk of developing PTSD and experience more severe PTSD symptoms than men. The increased vulnerability of women is not yet fully understood and can be attributed to a combination of biological and psychosocial factors. Despite the higher risk, women and female-specific risk factors are underrepresented in relevant research, a gender data gap exists. Men are more likely to experience accidental and weapon-related trauma and women are more likely to be exposed to sexual violence. While women more frequently suffer from comorbid anxiety and affective disorders, PTSD in men occurs more frequently in combination with substance abuse. Men with PTSD are less likely to seek psychotherapeutic help than women and benefit less from evidence-based trauma-focused interventions.

**Discussion:**

The differential consideration of biological and psychosocial factors is crucial to understanding sex and gender differences in PTSD. Sex and gender-sensitive approaches in diagnostics and treatment as well as the consideration of sex/gender beyond binary classifications can help to close knowledge gaps and enable more targeted care.

Obwohl Männer häufiger traumatische Ereignisse erleben, haben Frauen ein höheres Risiko, an einer posttraumatischen Belastungsstörung (PTBS) zu erkranken. Männer profitieren hingegen weniger von evidenzbasierten Interventionen. Geschlechterunterschiede prägen Entstehung, Verlauf und Therapie – werden aber in Forschung und Praxis noch zu wenig berücksichtigt. Der Beitrag zeigt, inwiefern geschlechtersensible Ansätze zu einer wirksameren Versorgung der posttraumatischen Belastungsstörung beitragen können.

## Geschlechterunterschiede in Traumaexposition

**Abb. 1:**
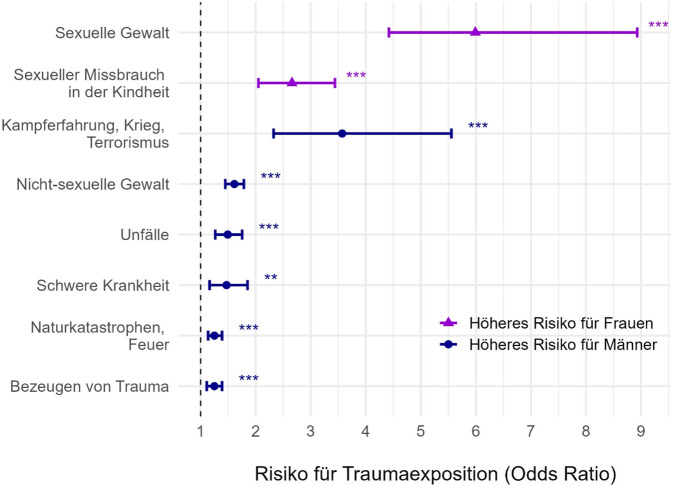
Geschlechtsunterschiede in der Traumaexposition nach Traumatyp. (Eigene Abbildung basierend auf Daten von Tolin und Foa [43]). *Violett* dargestellte Odds Ratios (OR) beschreiben ein höheres Risiko von Frauen im Vergleich zu Männern (Referenzgruppe). *Blau* dargestellte OR beschreiben ein höheres Risiko für Männer im Vergleich zu Frauen (Referenzgruppe). *p < .05, **p < .01, ***p < .001

Die Prävalenz traumatischer Ereignisse ist hoch: In Europa berichteten 2012 67,0 % der Männer und 60,5 % der Frauen, mindestens ein Trauma in ihrem Leben erlebt zu haben [44]. Die höhere Prävalenz von Traumata bei Männern ist auch international sowie metaanalytisch belegt [43]. Es finden sich zudem konsistente Unterschiede in der Art der traumatischen Erlebnisse. Während Männer häufiger körperlichen Angriffen, bewaffneten Kämpfen sowie Unfällen oder Bränden ausgesetzt sind, erleben Frauen häufiger sexuelle Übergriffe und Belästigung im Erwachsenenalter sowie sexuellen Missbrauch in der Kindheit ([35]; Abb. 1).

Interessanterweise deuten neuere epidemiologische Befunde auf weniger große Geschlechterunterschiede in der Häufigkeit erlebter Traumata hin [34]. Diese Ergebnisse werden aufgrund einer differenzierteren Erhebung von Traumata diskutiert, von denen insbesondere Frauen betroffen sind (z. B. Partnerschaftsgewalt, Stalking [34]). Hierfür spricht auch, dass bis zu 22 % aller Geburten die Kriterien für ein Trauma erfüllen [45]. Trotz dieser hohen Prävalenz sind traumatische Geburten in den etablierten Traumachecklisten nicht enthalten, wodurch die Prävalenz von Traumata bei Frauen unterschätzt wird.

## Geschlechterunterschiede im PTBS-Risiko

Trotz der hohen Prävalenz von Traumata [3], entwickeln nur wenige Betroffene anschließend eine PTBS. Weltweit erkranken etwa 6 % der Menschen, die ein Trauma erlebt haben, im Verlauf ihres Lebens an einer PTBS [28]. In Deutschland liegt die 1‑Monats-Prävalenz für PTBS in der Allgemeinbevölkerung bei 1–3 % [32].

Die PTBS zählt zu den psychischen Störungen mit den größten Geschlechterunterschieden. Frauen haben ein zwei- bis dreifach höheres Risiko als Männer, an einer PTBS zu erkranken [40]. Epidemiologische Befunde aus den Jahren 2009 bis 2012 schätzen die 12-Monats-Prävalenz von PTBS in Deutschland auf 0,9 % bei Männern und 3,6 % bei Frauen [23]. Bevölkerungsrepräsentative Daten, welche die geopolitischen Entwicklungen der vergangenen Jahre berücksichtigen, liegen für Deutschland derzeit nicht vor. Länderübergreifende Daten aus 15 Industrie- und Entwicklungsländern zeigen, dass die Wahrscheinlichkeit für eine PTBS bei Frauen weltweit (Odds Ratio =) 1,3- bis 6,4-mal so hoch ist wie bei Männern [40]. Dies weist auf eine differenzielle Ätiologie hin, die biologische und psychosoziale Faktoren umfasst. Gleichzeitig sind Frauen im Vergleich zu Männern in der bisherigen Forschung deutlich unterrepräsentiert [16]: Wir finden ein Gender-Data-Gap.

Ein Blick auf die zeitliche Ausdifferenzierung zeigt, dass das höhere PTBS-Risiko von Frauen bereits einen Monat nach Traumaexposition besteht – also zum frühesten Zeitpunkt, zu dem eine PTBS-Diagnose möglich ist. In der Folge scheinen die Unterschiede in Prävalenz und Schweregrad relativ stabil zu bleiben, sowohl bezüglich des zeitlichen Abstands zum Trauma [16] als auch über die Lebensspanne [48].

## Geschlechtersensible Diagnostik der PTBS

Neben dem erhöhten Risiko für eine PTBS-Diagnose leiden Frauen weiterhin unter schwerwiegenderen PTBS-Symptomen als Männer [16, 43]. Ein Blick auf die PTBS-Symptomcluster (d. h. Wiedererleben, Vermeidungsverhalten, Übererregbarkeit sowie negative Kognitionen und Stimmung) zeigt, dass sich der größere Leidensdruck von Frauen über alle vier im Diagnostic and Statistical Manual of Mental Disorders 5 (DSM-5) definierten Symptomkategorien erstreckt [21].

Trotz der höheren PTBS-Symptomschwere bei Frauen zeigen konfirmatorische Faktorenanalysen Messinvarianz zwischen Frauen und Männern [5]. Das bedeutet, dass die Prävalenzunterschiede nicht auf die Messmethodik zurückzuführen sind. Darüber hinaus zeigen Netzwerkanalysen, dass PTBS-Symptome bei Frauen und Männern in ähnlicher Weise miteinander verbunden sind [38]. Basierend auf der derzeit verfügbaren Evidenz gibt es daher keine Hinweise darauf, dass sich die verfügbaren Diagnostik-Instrumente hinsichtlich ihrer Eignung für Männer oder Frauen unterscheiden.

In Bezug auf die Erfassung traumatischer Erlebnisse ergibt sich jedoch eine Besonderheit. Traumata, von denen insbesondere Frauen betroffen sind (z. B. Partnerschaftsgewalt, Stalking, traumatische Geburten) werden über etablierte Traumachecklisten häufig nicht adäquat erfasst und daher übersehen. Die etablierten Fragebögen sollten in Forschung und Praxis standardmäßig um diese Traumatypen ergänzt werden.

## Geschlechterunterschiede in der komplexen PTBS

Die komplexe PTBS (kPTBS) wurde im International Statistical Classification of Diseases and Related Health Problems 11 (ICD-11) als neue Diagnose aufgenommen. Ergänzend zu den allgemeinen PTBS-Kriterien umfasst die kPTBS drei weitere Kriterien in Bezug auf die Selbstorganisation:

- eine Störung der Emotionsregulation,
- ein negatives Selbstkonzept sowie
- interpersonelle Schwierigkeiten.

Weiterhin wird die kPTBS auf anhaltende oder wiederholte, häufig interpersonelle Traumata zurückgeführt. Im Gegensatz zur PTBS zeigen sich bezüglich der kPTBS keine eindeutigen Prävalenzunterschiede zwischen Frauen und Männern [31]. Dieser Befund steht im Einklang mit Studien, die zeigen, dass Geschlechterunterschiede bei einem hohen Ausmaß an interpersoneller Traumatisierung geringer ausfallen [20]. Dies deutet darauf hin, dass geschlechtsbezogene Prozesse unter anhaltender, schwerwiegender Traumatisierung weniger relevant werden.

## Geschlechterunterschiede in Komorbiditäten

Die PTBS ist eine Störung mit hoher Komorbidität: Über 60 % der PTBS-Patient*innen leiden ebenfalls an einer affektiven, Angst- oder Substanzkonsumstörung [35]. Männer mit PTBS leiden häufiger an einer komorbiden Substanzkonsumstörung, während die PTBS bei Frauen häufiger mit komorbiden Depressionen oder Angststörungen einhergeht [22]. Hinsichtlich somatischer Komorbiditäten wird die PTBS mit einem erhöhten Risiko für Typ-2-Diabetes und Herz-Kreislauf-Erkrankungen assoziiert, wobei diese Zusammenhänge bei Frauen noch größer zu sein scheinen als bei Männern [14, 36]. Während der Schwangerschaft erhöht die PTBS das Risiko für Komplikationen wie Frühgeburten oder ein niedriges Geburtsgewicht [7, 41]. Nach der Geburt können Beeinträchtigungen in der funktionalen und emotionalen Verfügbarkeit wiederum das Risiko für Bindungsschwierigkeiten und für spätere Psychopathologien des Kindes erhöhen [10].

## Geschlechterunterschiede in der Pathogenese

Im Risikofaktor weibliches Geschlecht subsummiert sich ein komplexes Zusammenspiel biologischer („sex“), psychologischer und sozialer („gender“) Prozesse [6]. Dabei sind der relative Einfluss sex- und genderbezogener Prozesse sowie Ausmaß und Richtung der Interaktionen zwischen ihnen noch nicht ausreichend untersucht. Insbesondere mangelt es bislang an Studien, die beide Prozesse gemeinsam betrachten [18].

**Abb. 2:**
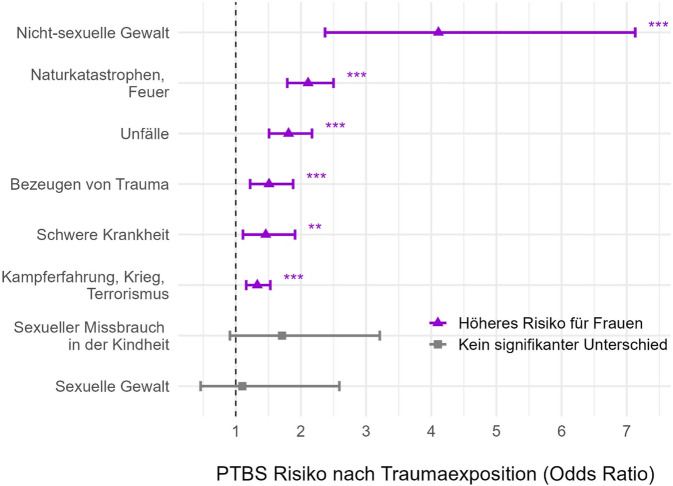
Geschlechtsunterschiede im Risiko für posttraumatische Belastungsstörungen (*PTBS*) nach Traumatyp. (Eigene Abbildung basierend auf Daten von Tolin und Foa [43]). Sowohl *violett* als auch *grau* dargestellte Odds Ratios beschreiben das Risiko für Frauen im Vergleich zu Männern (Referenzgruppe). *p < .05, **p < .01, ***p < .001

*Genderbezogene Traumatisierungsmuster* (Abb. 1) spielen eine entscheidende Rolle: Da sexuelle Gewalt mit einem höheren PTBS-Risiko einhergeht als z. B. Unfälle, trägt die höhere Prävalenz dieser Traumata bei Frauen wesentlich zu ihrem erhöhten PTBS-Risiko bei. Gleichzeitig kann dieser Faktor alleine das höhere PTBS-Risiko von Frauen nicht ausreichend erklären: So weisen Männer, die sexuelle Gewalt erlebt haben, ein vergleichbares PTBS-Risiko auf wie Frauen mit sexueller Gewaltexposition. Für alle weiteren Traumatypen hingegen zeigen Frauen ein erhöhtes PTBS-Risiko (Abb. 2).

Hinsichtlich weiterer Faktoren findet eine aktuelle metaanalytische Untersuchung vorwiegend ähnliche Schutz- und Risikofaktoren für Frauen und Männer [15]. Dies deutet darauf hin, dass sich die grundlegenden Prozesse für Männer und Frauen ähneln. Gleichzeitig scheinen Frauen von bestimmten, besonders einflussreichen Risikofaktoren besonders häufig betroffen zu sein [15]. Neben der höheren Prävalenz sexueller Gewalt (s. oben) scheint hier die unmittelbare Stressreaktion nach einem traumatischen Ereignis ein weiterer wichtiger Faktor zu sein. Frauen zeigen während und unmittelbar nach der Exposition stärkere akute psychologische Stresssymptome, die ein bedeutender Prädiktor für die Entwicklung einer PTBS sind [8, 15]. Mit Blick auf biologische, d. h. *sexbezogene Aspekte* deuten genetische Studien auf eine höhere Heritabilität bei Frauen als bei Männern hin [2]. Auch die Tatsache, dass Geschlechterunterschiede bei der PTBS sich mit Beginn der Pubertät – einer hormonellen Umbruchsphase – manifestieren, wird als Hinweis für die Relevanz biologischer Prozesse gesehen. Ein vertiefender Überblick über Geschlechteraspekte in der biologischen Stressreaktion sowie die Rolle der Geschlechtshormone Östradiol und Progesteron ist in Anhang 1 (Zusatzmaterial online) dargestellt.

Neben *geschlechtsabhängigen Faktoren* findet sich also auch eine Reihe *geschlechtsspezifischer Faktoren*, die ausschließlich Personen mit (biologisch) weiblichem Geschlecht betreffen – etwa die hormonellen Schwankungen im Menstruationszyklus. Traumata während des Gebärens betreffen ebenfalls ausschließlich (biologisch) weibliche Personen [19]. Gleichzeitig finden sich im Bereich der reproduktiven Gesundheit von Frauen nach wie vor große Forschungslücken und der Einfluss dieser Faktoren auf eine spezifisch weibliche Ätiologie ist bisher noch nicht ausreichend verstanden. Demgegenüber sind männlich-spezifische Risikofaktoren bisher kaum identifiziert. Zusammenhänge mit höheren PTBS-Symptomen zeigen sich jedoch für traditionelle Männlichkeitsideologien [24].

Zusammengefasst lässt sich festhalten, dass trotz der gut belegten Geschlechterunterschiede in Prävalenz und Schweregrad der PTBS ein umfassendes und differenziertes Verständnis der zugrunde liegenden Prozesse bislang fehlt [6]. Weitere Forschung ist nötig, um zu besser verstehen wie *Sex* und *Gender *sowie deren Interaktion psychische Gesundheit nach Traumaexposition beeinflussen.

## Geschlechterunterschiede in der Therapie der PTBS

Studien zur Wirksamkeit von Psychotherapien weisen mehrheitlich auf ungünstigere Behandlungsverläufe bei Männern als bei Frauen hin. Im Einklang mit allgemeinen Befunden zur Inanspruchnahme von Gesundheitsleistungen [12] zeigt sich auch bei der PTBS, dass betroffene Männer seltener psychotherapeutische Hilfe aufsuchen als Frauen [30]. Tun sie es doch, so profitieren sie weniger als Frauen von traumafokussierten Interventionen – der leitliniengerechten Behandlung erster Wahl [25]. Die Gründe hierfür sind bislang noch weitestgehend unklar. Vermutet werden u. a. Geschlechterunterschiede im Extinktionslernen sowie im Emotionsausdruck [25]. So falle es Männern häufig schwerer, Emotionen zuzulassen und auszudrücken, was die Wirksamkeit der Interventionen beeinträchtigen könnte [25]. Diese Vermutungen sind jedoch bisher nicht in Behandlungsstudien belegt und es bedarf dringend belastbarer Studien. Behandler*innen sollten im therapeutischen Kontakt individuelle genderbezogene Selbstkonzepte sensibel berücksichtigen und Stereotypisierung vermeiden.

Obwohl die klinischen Leitlinien die traumafokussierte Psychotherapie als Behandlung erster Wahl hervorheben [39], werden Personen mit PTBS häufig Psychopharmaka verschrieben, wobei dies häufiger auf Frauen als auf Männer zutrifft (OR = 1,62 [17]). Infobox 1 fasst die wichtigsten Geschlechterunterschiede bei der PTBS zusammen.

## Geschlecht und Diversität

Trotz einiger Fortschritte bestehen nach wie vor erhebliche Lücken in Bezug auf Diversität und strukturelle Gerechtigkeit in der PTBS-Forschung. Neben dem bereits benannten Gender-Data-Gap und der Unterbeforschung weiblicher Risikofaktoren [15, 16] besteht weiteres Potenzial, die Forschung geschlechtersensibler zu gestalten: In der Mehrzahl der aktuellen Studien werden weder Erhebungsmethoden zu Sex und Gender berichtet, noch diese Konzepte klar voneinander getrennt [16]. Auch die vielfältigen Facetten von Gender (z. B. Genderidentität, Genderrollen, Genderausdruck etc.) und Sex (z. B. Hormonlevel, Zellcharakteristika, Genotyp, etc.) sind in der aktuellen Forschung nicht ausreichend berücksichtigt. Problematisch ist weiterhin die ungenügende Berücksichtigung von Personen, die sich außerhalb binärer Geschlechterkategorien identifizieren. Obwohl Angehörige geschlechtlicher Minderheiten ein besonders hohes Risiko für interpersonelle Traumata und PTBS aufweisen [33], bestehen erhebliche Evidenzlücken, z. B. mit Blick auf Komorbiditäten, Therapieerfolg oder Prävalenz der kPTBS: Es ist unklar, ob und wie sich Geschlechterminderheiten diesbezüglich von Cis- und Endo-Personen sowie innerhalb der diversen Gruppe an Geschlechterminderheiten (z. B. nonbinäre und Trans*Personen) unterscheiden. Eine differenziertere und inklusivere Erhebung von Sex und Gender ist also essenziell, um eine adäquate Abbildung aller betroffenen Gruppen zu gewährleisten.

Ein ähnliches Problem zeigt sich in Bezug auf kulturelle Diversität. Zwar ist das erhöhte PTBS-Risiko bei Frauen im Vergleich zu Männern durch Metaanalysen gut belegt, dennoch zeigt sich eine erhebliche Heterogenität zwischen den Studien. So variieren Geschlechterunterschiede teils deutlich, wenn Analysen nach ethnischer Zugehörigkeit differenziert werden [37]. In kriegsbetroffenen Populationen wurden hingegen kaum oder keine Unterschiede zwischen Frauen und Männern festgestellt [46] – ein Befund, der sich auch in den ähnlichen geschlechtsbezogenen Risiken für die kPTBS widerspiegelt. Auch hier fehlt es an repräsentativen Daten, um die zugrunde liegenden Prozesse besser zu verstehen. Um diese Lücken zu füllen, braucht es dringend diversitäts- und kultursensible Forschungsperspektiven, die über binäre Geschlechterkategorien hinausgehen und auch intersektionale Aspekte berücksichtigen.

### Infobox 1 Geschlechterunterschiede in der posttraumatischen Belastungsstörung (PTBS)

- Prävalenz und Schweregrad
  - Frauen leiden häufiger an einer PTBS als Männer
  - Frauen leiden unter schwerwiegenderen PTBS-Symptomen als Männer
  - Angehörige von Geschlechterminderheiten weisen ein besonders hohes PTBS-Risiko auf
- Traumaexposition
  - Männer sind häufiger körperlichen Angriffen, bewaffneten Kämpfen, Unfällen oder Bränden ausgesetzt als Frauen
  - Frauen erleben im Vergleich zu Männern häufiger sexuelle Übergriffe und Belästigung im Erwachsenenalter sowie sexuellen Missbrauch in der Kindheit
  - Angehörige von Geschlechterminderheiten weisen ein besonders hohes Risiko für interpersonelle Traumatisierung auf
- Risikofaktoren
  - Insgesamt zeigen sich ähnliche Schutz- und Risikofaktoren für Männer und Frauen
  - Besonders einflussreiche Risikofaktoren, z. B. sexuelle Traumatisierung, akute Stressreaktionen, sind bei Frauen stärker ausgeprägt als bei Männern
- Komorbiditäten
  - Frauen leiden im Vergleich zu Männern häufiger unter komorbiden affektiven und Angststörungen
  - Männer leiden im Vergleich zu Frauen häufiger unter komorbiden Substanzkonsumstörungen
- Behandlung und Praxis
  - Männer mit einer PTBS nehmen seltener psychotherapeutische Hilfe in Anspruch als Frauen
  - Männer profitieren im Vergleich zu Frauen weniger von traumafokussierter Psychotherapie, der leitliniengerechten Behandlung erster Wahl
  - Frauen mit PTBS werden häufiger Psychopharmaka verordnet als Männern

## Fazit für die Praxis

- Obwohl das erhöhte Risiko für posttraumatische Belastungsstörungen (PTBS) für Frauen schon lange bekannt ist, sind die Ursachen hierfür nicht ausreichend erforscht. Ein vertieftes Verständnis der zugrunde liegenden biologischen, psychologischen und sozialen Einflussfaktoren ist essenziell, um geschlechtersensible und individualisierte Präventions- und Therapiekonzepte zu entwickeln und zu implementieren.
- Trotz der höheren PTBS-Prävalenzen bei Frauen sollten Behandelnde unabhängig vom Geschlecht auf Anzeichen einer PTBS achten und Stereotypisierungen vermeiden.
- Bei Betroffenen sollten mögliche somatische (z. B. Diabetes, Herz-Kreislauf-Erkrankungen) und psychische (z. B. Depression, Substanzmissbrauch) Komorbiditäten abgeklärt werden. Gleichzeitig sollte bei Störungsbildern wie z. B. einer Substanzkonsumstörung eine mögliche zugrunde liegende PTBS abgeklärt werden.
- Traumata, von denen insbesondere Frauen betroffen sind (z. B. Stalking, Partnerschaftsgewalt oder Traumatisierung unter der Geburt), werden in Forschung und Praxis häufig übersehen und sollten zukünftig systematisch erfasst werden.

## Supplementary Information

Geschlechteraspekte in der biologischen Stressreaktion
