# Supplementary material for: Sex and gender differences in posttraumatic stress disorder: current evidence on etiology, trajectory and treatment
Source: Nervenarzt. 2025 Oct 6;97(1):34–41. [Article in German] doi: 10.1007/s00115-025-01907-6 (PMC12808190; doi:10.1007/s00115-025-01907-6)
Supplement: Supplementary file 1 — Geschlechteraspekte in der biologischen Stressreaktion [file 115_2025_1907_MOESM1_ESM.pdf]

Anhang zu

Geschlechterunterschiede bei der Posttraumatischen Belastungsstörung: aktuelle Evidenz zu Entstehung, Verlauf und Behandlung

Stephanie Haering, Caroline Meyer, Christine Knaevelsrud, Sinha Engel

Anhang 1 Geschlechteraspekte in der biologischen Stressreaktion

Neben einer hohen subjektiven Stressreaktion, die prädiktiv für die Entwicklung von PTBS-Symptomen ist, spielt auch die biologische Stressreaktion eine Rolle. In einer Anwendung des „building block-Effekts“, der kumulative Traumatisierung als Risikofaktor für eine PTBS postuliert, verweisen Steudte-Schmiedgen et al. [8] auf eine reduzierte Ausschüttung des Stresshormons Kortisol als zugrundeliegenden Prozess. Spezifisch wird angenommen, dass die Hypothalamus-Hypophysen-Nebennierenrinden-Achse (HHNA) auf ein Trauma reaktiv – also mit erhöhter Kortisolausschüttung – reagiert, die basale Aktivität der HHNA mit kumulativem traumatischen Stress jedoch zunehmend abnimmt, was einen Risikofaktor für die PTBS darstellt. Obwohl die genauen Interaktionen zwischen der HHNA mit ihrem Endprodukt Kortisol und der Hypothalamus-Hypophysen-Gonaden-Achse (HPGA) mit ihren Endprodukten Östrogen und Progesteron (weibliche Geschlechtshormone) sowie Testosteron (männliches Geschlechtshormon) noch genauer verstanden werden müssen, gibt es bereits einige beeindruckende Befunde, zu ihrem Zusammenspiel bei traumatischem Stress. Grundsätzlich beeinflussen weibliche Geschlechtshormone sowohl die basale HHNA-Aktivität – mit höheren Kortisolkonzentrationen in der Follikelphase, verglichen mit der lutealen Phase des Menstruationszyklus [6] (REF)– als auch ihre Reaktivität, die wiederum in der lutealen Phase höher ist als in der Follikelphase [5]. In Bezug auf PTBS wird schon die Tatsache, dass Geschlechterunterschiede sich mit Beginn der Pubertät - einer hormonellen Umbruchsphase - manifestieren, als Hinweis für die Relevanz weiblicher Geschlechtshormone gesehen [4]. In klinischen Beobachtungsstudien konnte die Anwendung hormoneller Verhütungsmittel, welche die durch den Menstruationszyklus bedingte Schwankungen in den weiblichen Geschlechtshormonen unterdrücken, zum Zeitpunkt der Traumaexposition als Schutzfaktor identifiziert werden [2]. Die Frage nach dem Einfluss hormoneller

Schwankungen wurde bisher allerdings noch nicht ins Laborsetting übertragen, um diesen Prozess genauer zu untersuchen. Einige Laborstudien haben unter Anwendung des Trauma-Film-Paradigmas den Einfluss absoluter Östradiol- und Progesteronkonzentrationen untersucht, allerdings mit heterogenen Ergebnissen, die auf weiteren Forschungsbedarf hinweisen [3, 7]. Weibliche Geschlechtshormone beeinflussen nicht nur die Entstehung, sondern auch den Ausdruck von PTBS-Symptomen. So berichten PTBS-Patientinnen während der Lutealphase, die durch einen starken Anstieg und anschließenden Abfall von Östradiol- und Progesteronkonzentrationen gekennzeichnet ist, deutlich mehr Flashbacks [1].

Referenzen

1. Bryant RA, Felmingham KL, Silove D et al (2011) The association between menstrual cycle and traumatic memories. Journal of Affective Disorders 131:398–401. https://doi.org/10.1016/j.jad.2010.10.049

2. Engel S, Van Zuiden M, Frijling JL et al (2019) Patterns of Recovery From Early Posttraumatic Stress Symptoms After a Preventive Intervention With Oxytocin: Hormonal Contraception Use Is a Prognostic Factor. Biological Psychiatry 85:e71–e73. https://doi.org/10.1016/j.biopsych.2019.01.014

3. Franke LK, Miedl SF, Danböck SK et al (2022) Estradiol during (analogue-)trauma: Risk- or protective factor for intrusive re-experiencing? Psychoneuroendocrinology 143:105819. https://doi.org/10.1016/j.psyneuen.2022.105819

4. Garza K, Jovanovic T (2017) Impact of Gender on Child and Adolescent PTSD. Curr Psychiatry Rep 19:87. https://doi.org/10.1007/s11920-017-0830-6

5. Klusmann H, Luecking N, Engel S et al (2023) Menstrual cycle-related changes in HPA axis reactivity to acute psychosocial and physiological stressors – A systematic review and meta-analysis of longitudinal studies. Neuroscience & Biobehavioral Reviews 150:105212. https://doi.org/10.1016/j.neubiorev.2023.105212

6. Klusmann H, Schulze L, Engel S et al (2022) HPA axis activity across the menstrual cycle - a systematic review and meta-analysis of longitudinal studies. Frontiers in Neuroendocrinology 66:100998. https://doi.org/10.1016/j.yfrne.2022.100998

7. Krinke E, Held U, Steigmiller K et al (2022) Sex hormones and cortisol during experimental trauma memory consolidation: Prospective association with intrusive memories. European Journal of Psychotraumatology 13:2040818. https://doi.org/10.1080/20008198.2022.2040818

8. Steudte-Schmiedgen S, Kirschbaum C, Alexander N, Stalder T (2016) An integrative model linking traumatization, cortisol dysregulation and posttraumatic stress disorder: Insight from recent hair cortisol findings. Neuroscience & Biobehavioral Reviews 69:124–135. https://doi.org/10.1016/j.neubiorev.2016.07.015
